# Supplementary material for: Residues and Dietary Risk Assessment of Prohexadione-Ca and Uniconazole in Oryza sativa L. and Citrus reticulata Blanco by Liquid Chromatography-Tandem Mass Spectrometry
Source: Molecules. 2023 Mar 13;28(6):2611. doi: 10.3390/molecules28062611 (PMC10059882; doi:10.3390/molecules28062611)
Supplement: Supplementary file 1 [file molecules-28-02611-s001.zip › molecules-2260253-supplementary.pdf]

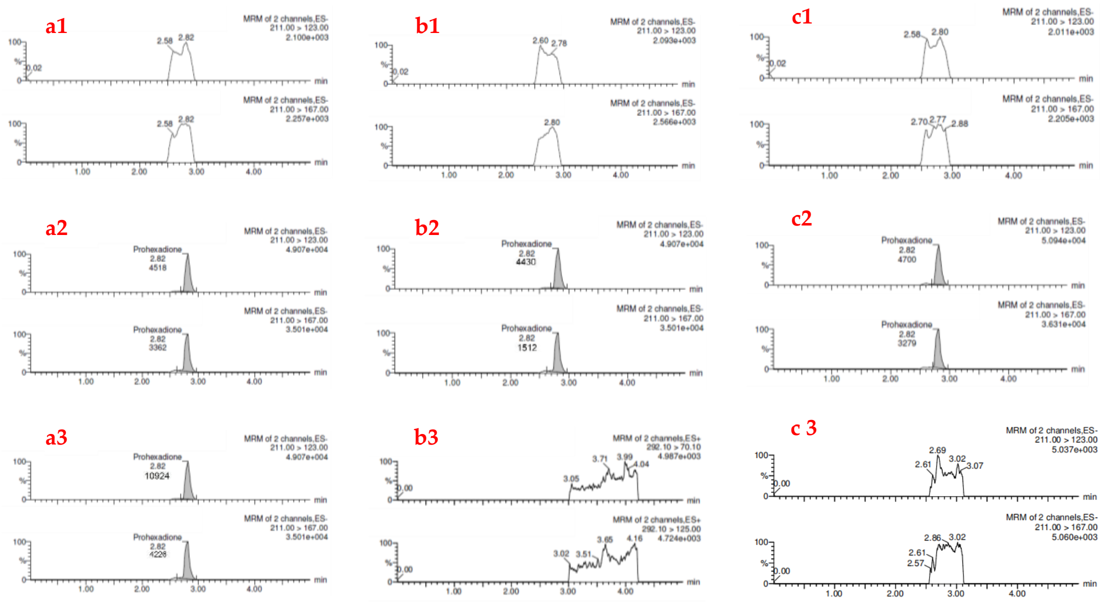

Figure S1. Typical LC-MS/MS MRM chromatograms of prohexadione and prohexadione-Ca: (a1) solvent blank, (a2) 0.01 mg/L solvent standard, (a3) 0.01 mg/L matrix-matched standard (brown rice); (b1) brown rice blank, (b2) brown rice spiked at 0.05 mg/kg prohexadione, (b3) brown rice sample collected at 112 d; (c1) whole citrus fruit blank, (c2) whole citrus fruit spiked at 0.05 mg/kg prohexadione, (c3) whole citrus fruit sample collected at 150 d.

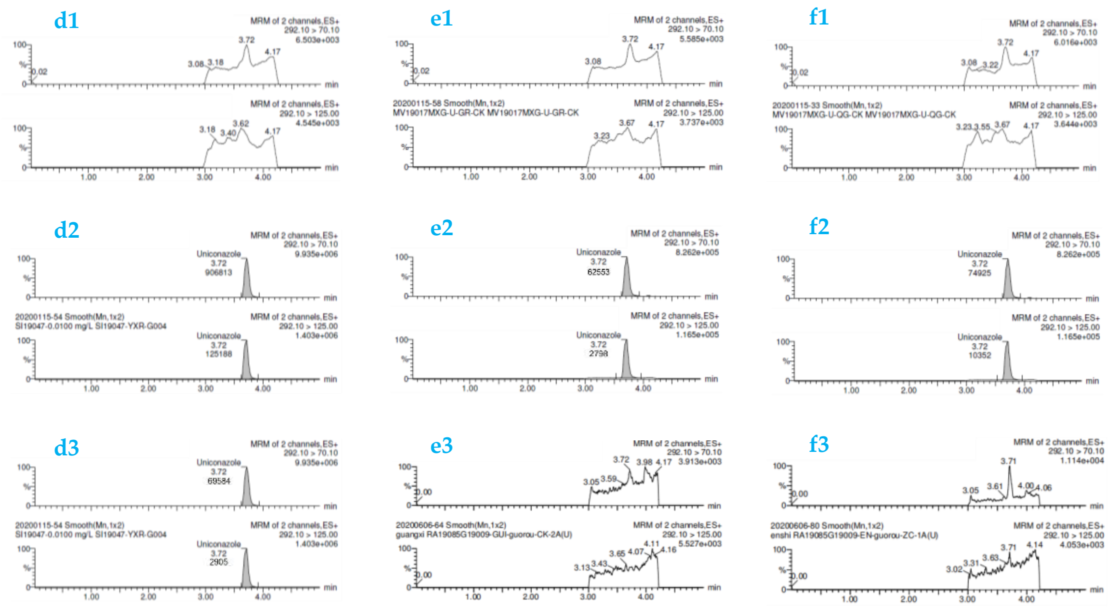

Figure S2. Typical LC-MS/MS MRM chromatograms of uniconazole: (d1) solvent blank, (d2) 0.01 mg/L solvent standard, (d3) 0.05 mg/L matrix-matched standard (brown rice); (e1) brown rice blank, (e2) brown rice spiked at 0.01 mg/kg uniconazole, (e3) brown rice sample collected at 112 d; (f1) whole citrus fruit blank, (f2) whole citrus fruit spiked at 0.01 mg/kg uniconazole, (f3) whole citrus fruit sample collected at 150 d.

Table S1. Storage stability of prohexadione, prohexadione-Ca, and uniconazole in rice and citrus samples fortified at 0.5 mg/kg.

Matrix	Storage interval (d)	Residues remaining in stored samples (mg/kg; % degradation)		
		Prohexadione	Prohexadione–Ca	Uniconazole
Brown rice	0 (Initial)	0.445	0.516	0.501
	29	0.484 (-8.9%)	0.552 (-7.0%)	0.520 (3.8%)
	87	0.416 (-6.5%)	0.563 (-9.0%)	0.520 (3.8%)
	179	0.500 (-12.0%)	0.529 (-3.9%)	0.518 (3.3%)
Rice hull	0 (Initial)	0.455	0.520	0.487
	29	0.407 (11.0%)	0.523 (-0.7%)	0.501 (2.9%)
	87	0.423 (7.1%)	0.434 (16.0%)	0.404 (-17.0%)
	179	0.370 (19.0%)	0.436 (16.0%)	0.536 (10.0%)
Rice straw	0 (Initial)	0.418	0.518	0.488
	29	0.373 (11.0%)	0.489 (5.6%)	0.576 (-18.0%)
	87	0.412 (1.4%)	0.531(-2.5%)	0.547 (-12.0%)
	179	0.420 (-0.6%)	0.612 (-18.0%)	0.566 (-16.0%)
Whole citrus fruit	0 (Initial)	0.512	0.448	0.528
	30	0.493 (3.7%)	0.420 (6.3%)	0.510 (3.5%)
	329	0.546 (-6.7%)	0.483 (-7.9%)	0.551 (-4.4%)
	380	0.478 (6.7%)	0.511 (-14.0%)	0.448 (15.0%)
Citrus pulp	0 (Initial)	0.475	0.458	0.513
	30	0.496 (-4.5%)	0.427 (6.7%)	0.511 (0.4%)
	329	0.542 (-14.0%)	0.498 (-8.7%)	0.522 (-1.8%)
	380	0.490 (-3.1%)	0.505 (-10.0%)	0.461 (10.0%)

Table S2. Field collection information for rice and citrus samples from 12 representative sites.

sample	Site	variety	Time	Harvest interval (days)
Rice	Hangzhou, Zhejiang	Yongyou 4550	July 31, 2020 - October 28, 2020	89
	Jiujiang, Jiangxi	Jiu'er Youhua Zhan	August 29, 2020 - November 1, 2020	64
	Shenyang, Liaoning	Shengfeng 88	June 18, 2020 - October 8, 2020	112
	Jianshi, Hubei	Shenyou 9716	July 23, 2020 - October 14, 2020	83
	Changsha, Hunan	Liangyou 1207	July 24, 2020 - October 24, 2020	92
	Nanning, Guangxi	Y Liangyou 2	September 16, 2020 - November 17, 2020	62
	Niangping, Chongqing	Fanyou 709	June 13, 2020 - September 15, 2020	94
	Hefei, Anhui	Zaoxian 802	July 22, 2020 - September 30, 2020	70
	Guiyang, Guizhou	Yun Liangyou Simiao	June 25, 2020 - October 8, 2020	105
	Hohhot, Inner Mongolia	Jinhuan Xiang 3	July 7, 2020 - October 9, 2020	94
	Fu'an, Fujian	Shen Liangyou 5814	June 4, 2020 - August 8, 2020	65
	Zhaoqing, Guangdong	Meixiang Zhan	September 2, 2020 - November 9, 2020	68
Citrus	Jinhua, Zhejiang	Gongchuan	May 29, 2019 - November 12, 2019	167
	Gaoan, Jiangxi	Wenzhou honey mandarin	June 5, 2019 - November 2, 2019	150
	Wuhan, Hubei	Jinshui orange	May 13, 2019 - November 20, 2019	191
	Lichuang, Hubei	Wenzhou honey mandarin	June 2, 2019 - October 30, 2019	157
	Changsha, Hunan	Shaju orange	June 3, 2019 - December 11, 2019	150
	Nanning, Guangxi	Hongjiang orange	June 3, 2019 - December 11, 2019	191
	Beibei, Chongqing	Navel orange	August 12, 2019 - January 24, 2020	165
	Chengdu, Sichuang	Haruken	June 12, 2019 - December 20, 2019	191
	Qiannan, Guizhou	Niurouhong	May 20, 2019 - November 7, 2019	171
	Fu'an, Fujian	Luju orange	June 4, 2019 - November 11, 2019	160
	Zhangzhou, Fujian	Lukan	June 29, 2019 - December 7, 2019	161
	Zhanjiang, Guangdong	Hongjiang orange	May 16, 2019 - October 20, 2019	157
